# The Circadian Clock—A Molecular Tool for Survival in Cyanobacteria

**DOI:** 10.3390/life10120365

**Published:** 2020-12-20

**Authors:** Pyonghwa Kim, Manpreet Kaur, Hye-In Jang, Yong-Ick Kim

**Affiliations:** 1 Department of Chemistry and Environmental Science, New Jersey Institute of Technology, Newark, NJ 07102, USA; pk479@njit.edu (P.K.); mk359@njit.edu (M.K.); 2 School of Cosmetic Science and Beauty Biotechnology, Semyung University, Jecheon 27136, Korea; 3 Institute for Brain and Neuroscience Research, New Jersey Institute of Technology, Newark, NJ 07102, USA

**Keywords:** circadian clock, circadian rhythm, cyanobacteria, KaiABC, SasA, CikA, RpaA

## Abstract

Cyanobacteria are photosynthetic organisms that are known to be responsible for oxygenating Earth’s early atmosphere. Having evolved to ensure optimal survival in the periodic light/dark cycle on this planet, their genetic codes are packed with various tools, including a sophisticated biological timekeeping system. Among the cyanobacteria is *Synechococcus elongatus* PCC 7942, the simplest clock-harboring organism with a powerful genetic tool that enabled the identification of its intricate timekeeping mechanism. The three central oscillator proteins—KaiA, KaiB, and KaiC—drive the 24 h cyclic gene expression rhythm of cyanobacteria, and the “ticking” of the oscillator can be reconstituted inside a test tube just by mixing the three recombinant proteins with ATP and Mg^2+^. Along with its biochemical resilience, the post-translational rhythm of the oscillation can be reset through sensing oxidized quinone, a metabolite that becomes abundant at the onset of darkness. In addition, the output components pick up the information from the central oscillator, tuning the physiological and behavioral patterns and enabling the organism to better cope with the cyclic environmental conditions. In this review, we highlight our understanding of the cyanobacterial circadian clock and discuss how it functions as a molecular chronometer that readies the host for predictable changes in its surroundings.

## 1. Introduction—The Free-Running Cyanobacterial Central Oscillator

As the Earth spins on its own axis with a 24 h periodicity as it revolves around the Sun, light and darkness take their turn, marking the start and end of a day respectively. Ever since life first appeared on this planet, organisms that have been constantly exposed to these periodic changes started taking advantage of them by developing a biological timekeeping mechanism. This is because the internal clock would provide a means to anticipate and prepare for the imminent sunrise or sunset and temperature fluctuations. Although this does not seem like much, the ability to keep time often becomes the difference between life and death, much more so in cyanobacteria, and it is no wonder why they developed such a sophisticated circadian clock as a mechanism to maintain such longevity on this planet.

**Figure 1:**
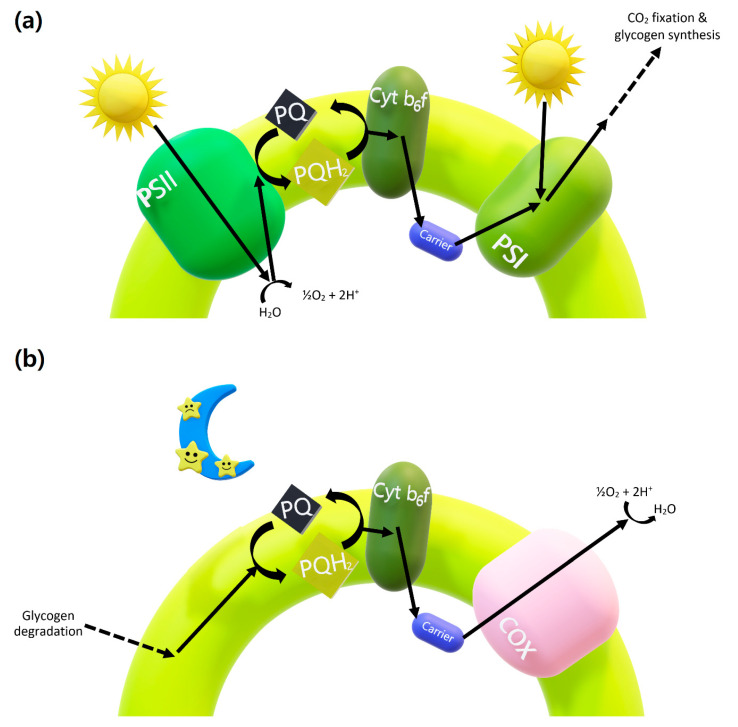
Simplified schematic diagram of photosynthesis and cellular respiration in the thylakoid membrane of cyanobacteria: (**a**) photosynthesis during the day and (**b**) cellular respiration at night. Black arrows represent the transfer of electrons. The transition from day to night causes the plastoquinone (PQ) pool to become acutely oxidized, and conversely, the transition to the day leads to its transient reduction. COX: cytochrome-c oxidase, Cyt b_6_f: cytochrome b_6_f, PQH_2_: plastoquinol, PSI/PSII: Photosystem I/II.

To put it simply, photosynthesis is the splitting of water using light energy to fix and store carbon, and respiration is the converse, the burning of the stored glycogen to keep up with the internal reducing power. As potential ancestors of modern-day plants, cyanobacteria are special in that they perform both photosynthesis and respiration in the same cellular compartment [1]. This means that the photosynthetic and respiratory electrons produced from light and in darkness, respectively, eventually pass through the same pathway, converging at the plastoquinone (PQ) pool (Figure 1). In addition, the cyanobacterial circadian clock temporally regulates glycogen synthesis and degradation to ensure that the energy storage and utilization take place at the right time for survival [2,3,4,5]. Cyanobacteria must run these two biochemically incompatible processes in a timely manner in order to survive, and this provides unique challenges for cyanobacteria in that not only the redox state must be carefully balanced and modulated [6] but also these opposite reactions must take turns. Otherwise, over-reduction leads to the inevitable generation of reactive oxygen species (ROS), which can destroy any cellular components that they come into contact with. It is no surprise that cyanobacteria managed to put the production of an ROS-mediating protein under the control of its circadian clock, since the ROS level peaks along with that of the photosynthetic activity when the influx of electrons becomes especially abundant [7]. On the other hand, overoxidation is most often the result of starvation. A strain of cyanobacteria with a defective clock output pathway quickly perishes at the onset of darkness, and it has been suggested that this may be due to its inability to make the necessary enzymes for breaking down glycogen in a timely manner as it runs out of energy to survive at night [2]. In sum, the cyanobacterial circadian clock controls the essential physiological processes to maximize their chance of survival, carefully maintaining a balanced endogenous redox state and ensuring the expression of proteins to temporally meet the cellular demands.

*Synechococcus elongatus* PCC 7942 (hereafter, *S. elongatus*) is a freshwater-living strain of cyanobacteria that clearly demonstrates the characteristics of the endogenous timekeeping mechanism. Perhaps, this single-celled prokaryote’s resilience can be attributed to its intricately designed circadian clock, which ensures maximum flexibility in its response to environmental cues, as well as robustness such that it can withstand cellular cacophony. In this genetically tractable species, a circadian promoter with luciferase gene was inserted in a neutral site such that its clock-controlled gene expression cycle can be easily detected as the bioluminescence rhythm of dim and bright [8]. Then, some mutants displaying arrhythmic phenotypes paved the way for identifying that *S. elongatus*’ circadian oscillator is composed of three clock genes: *kaiA*, *kaiB*, and *kaiC* [9].

Resembling two stacked donuts, KaiC is a hexameric protein, composed of a CI and CII domain [10]; the CI domain harbors ATPase activity, which oscillates with circadian periodicity [11], and the CII domain undergoes cyclic phosphorylation and dephosphorylation. KaiA promotes its daytime kinase activity, and KaiB induces its nighttime phosphatase activity. The Kai proteins do not require de novo synthesis of proteins to run [12], and more surprisingly, when the oscillator proteins are purified and mixed with adenosine triphosphate (ATP) and magnesium (Mg^2+^) in a test tube, they retain their post-translational activity, which “ticks” as long as ATP is present [13,14].

In the CII domain of KaiC, there is a protruding linear chain (residues 488–497) called the “A-loop,” which switches between two conformations: “buried” and “exposed” [15]. When its position is buried, KaiC undergoes dephosphorylation; conversely, KaiC autophosphorylates when the A-loop is exposed. KaiA’s role at this point is to bind the A-loop (hence the name) and make it stay exposed [16]. KaiB, on the other hand, binds to the CI domain of fully phosphorylated KaiC and sequesters KaiA away from the A-loop, preventing it from interacting with the loop and thus resulting in KaiC dephosphorylation [17,18].

**Figure 2:**
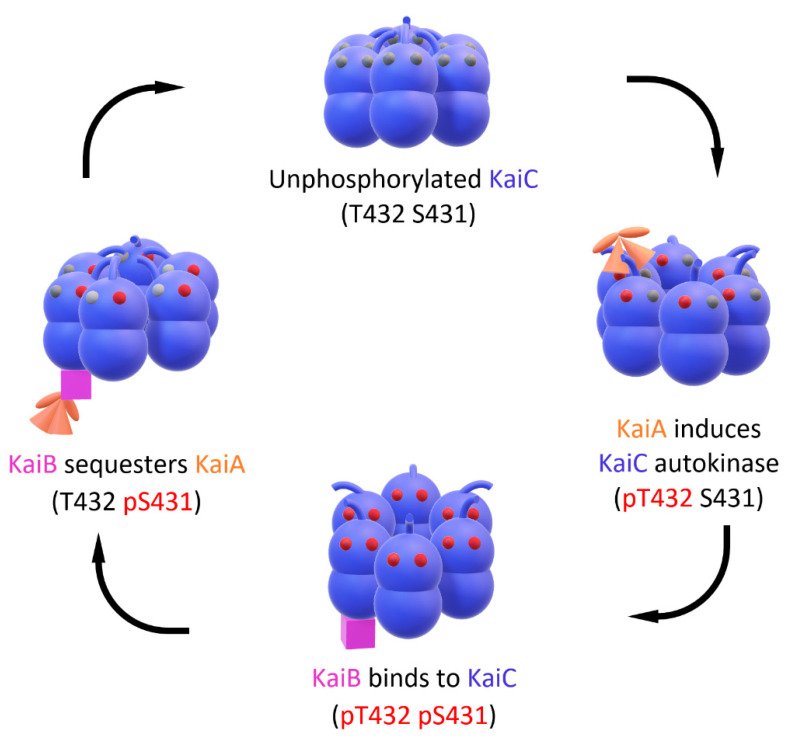
Ordered phosphorylation and dephosphorylation of KaiC. The initial unphosphorylated KaiC hexamer has the A-loops in their buried conformation by default. KaiA’s binding tethers the A-loops in their exposed conformation, initiating autokinase activity and phosphorylating T432 first. Then, KaiC reaches its fully phosphorylated state by phosphorylating its remaining S431. At this point, KaiC’s affinity for KaiB greatly increases due to their conformational changes [19,20]. KaiC–KaiB complexation ensues, sequestering KaiA away from the A-loop; the A-loops’ burial leads to the dephosphorylation of T432. Through further dephosphorylation, the KaiC hexamer returns to its unphosphorylated state.

One crucial property of a mechanical clock is to run “clockwise,” although it is not an easy feat for a biological clock to keep this unidirectionality [19,20]. There are two sites of phosphorylation in the CII domain of KaiC: S431 and T432 (Figure 2). When KaiA tethers to the exposed A-loop of KaiC and initiates kinase activity, T432 is phosphorylated first (pT432 S431). Then, S431 phosphorylation follows, and KaiC becomes fully phosphorylated (pT432 pS431). When KaiB binds KaiC and isolates KaiA from the A-loop, pT432 is dephosphorylated first (T432 pS431). Further dephosphorylation ensues, and KaiC returns to its unphosphorylated form (T432 S431) [21,22]. This extremely slow rate of ordered phosphorylation and dephosphorylation spans 24 h.

It is because of this free-running ability of the central oscillator that cyanobacteria can keep time, even in a constant environment, such as constant light, which makes sure that the internal clock is unperturbed by the environment [23]. Remarkably, the KaiC phosphorylation state stays the same among the divided cells while the cell undergoes this biologically “noisy” process of reproduction, maintaining the same phase of oscillation as a colony [24]. More surprising is KaiC’s ability to allosterically induce other monomers in the same hexameric complex to reach the same phosphorylation state through intersubunit shuffling [25]. It is not difficult to glean the prominence of timekeeping in cyanobacteria’s survival, considering their effort to synchronize the phase of the oscillator happens at both intercellular and intracellular levels.

In an in vivo study, a cyanobacterial culture that harbor the circadian clock, where its period matches with that of the light and dark cycle, showed better reproductive fitness and outcompeted other populations that had different clock periods [26]. Moreover, in a constant light environment, a strain that had a defective circadian clock outcompeted one with a properly functioning clock, meaning that a circadian clock is only useful in a rhythmically oscillating environment and even renders a disadvantage when exposed to a constant environment [27]. A beneficial adaptation for cyanobacteria to survive in a rhythmically oscillating environment on this planet was to develop an internal clock that has a 24 h periodicity.

However, although the in vivo mutational analyses provide an initial and basic insight on the phenotypic variation that results from a dysfunctional component, structural and functional analyses are needed in order to look at a more detailed picture of the biological timekeeping, phase resetting, and output signaling functions. By reconstituting the cyanobacterial circadian clock in vitro, we attempt to uncover detailed biochemical mechanisms behind the clockwork based on the most current structural data available in an attempt to answer how cyanobacteria manage to survive in various environmental niches.

**Figure 3:**
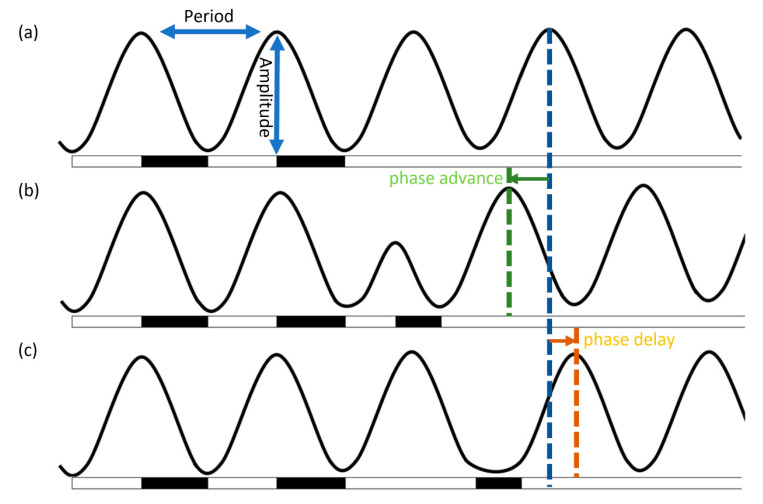
The entrainment of a circadian rhythm with a dark pulse. The white part on the *x*-axis represents light or daytime; the dark area represents either the application of a dark pulse in vivo or the application of ADP or oxidized quinone in vitro. The *y*-axis represents the circadian gene expression pattern or KaiC phosphorylation rhythm in cyanobacteria. (**a**) A free-running rhythm that still runs even under a continuous light condition. (**b**) A phase advance that is caused by a dark-related cue during the rising phase of the clock. (**c**) A phase delay that is caused by a dark-related cue during the falling phase.

The circadian oscillation of KaiC phosphorylation is readily detected in vitro via SDS-PAGE, wherein phosphorylated KaiC can be viewed as an upper triple band and dephosphorylated KaiC can be viewed as a lower single band on the gel [28]. The ratio of phosphorylated KaiC to total KaiC is determined by the densitometry of each band, and the ratio is plotted as a function of the incubation time. Ideally, the graph produced should represent the self-running 24 h oscillations of circadian rhythm (Figure 3a) [29].

## 2. Entrainment through the Input Pathway

A mechanical clock has hands that come back to the same spot with a certain periodicity. *S. elongatus*’ circadian clock, with its unusually high level of intricacy, seems to be equipped with this ability. Not only that, the time frame of natural competence can be shorter if the period of darkness becomes shorter, which is a condition that mimics longer days; the converse is also true for shorter days [30]. In addition, occasional cloudiness affects the gene expression pattern without affecting the KaiC phosphorylation profile [5]. These findings account for cyanobacteria’s adaptation to the seasonal variance. Cyanobacteria do not have to deal with an extreme clock misalignment, such as in the case of jetlag.

However, even the most accurate clock needs constant calibrations, and for that, a mechanical clock has a knob that can be turned to adjust to a new environmental time. Most organisms need to adjust their clock every day to maintain their internal physiological and behavioral activities in synchrony with the diurnal day/night cycle. Thus, one of the most crucial properties of the circadian clock is its ability to be entrained in response to an environmental cue. For cyanobacteria as well, the circadian clock’s free-running period of oscillation is not always exactly 24 h. In order to guarantee survival in an oscillating environment, the temporal division of the two metabolic reactions must be able to constantly synchronize to the environmental time.

Applying a dark period for 4 to 5 h (hereafter, a dark pulse) to a cyanobacterial culture causes the phase of the circadian oscillation to shift, and both a phase advance and delay can be generated depending on the time point of application (Figure 3b,c) [31]. How the information about light or darkness is passed down to the circadian clock and how cyanobacteria quickly shift their own phase in response to the environmental stimulus has intrigued a handful of scientists, although no direct light detection mechanism has been identified in cyanobacteria [32]. However, it was found that they use an indirect method of sensing the abundance of metabolites as a proxy for light/dark. Varying concentrations of ATP and adenosine diphosphate (ADP) shifted the circadian rhythm, both in vivo and in vitro [33].

During the day, the endogenous concentration of ATP becomes higher due to the superfluous influx of photosynthetic electrons, and more substrates become available to induce KaiC phosphorylation. Conversely, at night, ADP concentration gradually builds up, and it competes with ATP for the same binding site on KaiC, inducing its dephosphorylation as a result. This process was mimicked both in vivo and in vitro, and the presence of higher ATP and ADP induced premature phosphorylation and dephosphorylation, respectively, shifting the phase as a result [33]. The phase shift pattern matched with that of the in vivo dark pulse application; therefore, ATP/ADP was considered as the metabolite, where its periodic abundance aligns the peaking of the oscillatory phosphorylation of KaiC to the correct phase of the environmental cycle.

Truly, the ability to become entrained with variation in ATP/ADP concentration is a remarkable discovery, but there must be some other mechanism that ensures cyanobacteria’s more acute response to a dark pulse since a dark-induced drop in the ATP/ADP ratio only becomes apparent after at least 2 h [33]. This is because, if the onset of darkness is directly related to survival, cyanobacteria must have a way to send this vital information about imminent darkness to the circadian clock at a faster rate. In addition, a recent study reported that cyanobacteria subjected to darkness experience only a slight reduction in energy charge [2], and cyanobacteria’s resilience can be attributed to its ability to maintain a steady-state level of reduced power. Therefore, some other pathway is needed to explain cyanobacteria’s rather quick response to the sudden absence of light.

As previously mentioned, both photosynthetic and respiratory electrons eventually flow through the PQ pool (Figure 1) and measuring the redox state of the PQ pool has shown a profile that is in agreement with this notion [34]. Cyanobacteria try to maintain a steady-state level of the redox state, but darkness quickly oxidizes its quinone pool. Applying oxidized quinone to cyanobacteria makes KaiA become inactivated in vivo [35,36,37,38]. To test this effect in a more isolated and detailed manner by mimicking it in vitro, oxidized quinone’s effect was tested in a standard oscillator mixture KaiABC [34]. In this study, KaiA became inactivated by oxidized quinone in vitro, and more surprising was KaiA’s time dependency regarding this response. This is because inactivated KaiA can no longer induce KaiC phosphorylation and it depends on the time point of quinone application. KaiA inactivation with oxidized quinone shifted the phase of the oscillation, causing premature dephosphorylation of KaiC and forcing the rhythm to oscillate early; thus, a phase advance occurs. However, an expected phase delay was not produced; therefore, an in vitro approach using oxidized quinone to mimic the in vivo dark pulse entrainment process remained somewhat incomplete at this point.

Commonly, a photosynthetic organism detects the environmental day/night transition through the use of photoreceptors, such as a phytochrome [39,40]. Furthermore, the initial discovery of a phytochrome-like protein in cyanobacteria suggested a possibility that this might be one of the input components that convey the vital information about light or darkness to the circadian oscillator [37]. This protein was named “circadian input kinase A” (CikA); however, while phytochromes normally bind a chromophore called bilin in other light-harvesting organisms, CikA seemed unresponsive to bilin [35].

A crystal structure of CikA bound to the KaiB–KaiC complex confirmed its common binding spot on KaiB [41]. Later, it turned out that CikA competes with KaiA for the same binding site on KaiB during the time when KaiC–KaiB complexation takes place [42]. Again, KaiB’s role as an oscillator component is to sequester KaiA away from the A-loop in such a way that the isolated KaiA cannot lock it in its exposed conformation, leading to KaiC dephosphorylation. Adding CikA in the in vitro mixture shortened the oscillatory KaiC phosphorylation [43], which is an effect that is like adding more KaiA [43]. In agreement with this finding, an ectopic expression of CikA in vivo showed period shortening [44]. The displaced KaiA can bind to the A-loop to activate the kinase activity of KaiC. This may be because KaiB cannot sequester KaiA when CikA is occupying the same spot, leading to premature KaiC phosphorylation.

**Figure 4:**
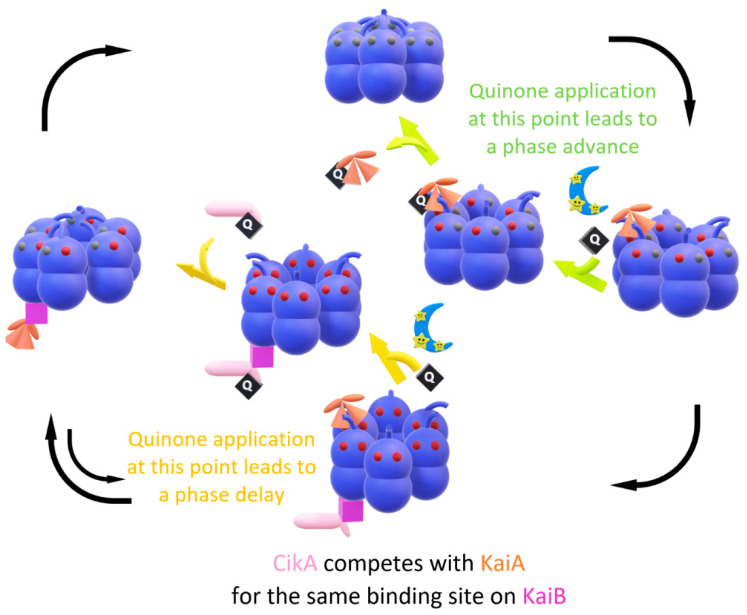
Schematic diagram of quinone entrainment. Oxidized quinone (“Q” in a black rhombus) becomes acutely abundant at night as a proxy for darkness. As KaiC autophosphorylation takes place, quinone application at this point inactivates KaiA, leading to premature dephosphorylation and a phase advance (lime arrows). On the other hand, in the in vitro mixture containing CikA and KaiABC, as fully phosphorylated KaiC enters the autodephosphorylating phase, quinone application at this point inactivates CikA, adding an extra time frame for the KaiA sequestration to take place, delaying the phase (yellow arrows).

KaiA is composed of a KaiC-interacting C-terminal domain, a linker, and a pseudoreceiver (PsR) domain, and it is this PsR domain where oxidized quinone binds and inactivates KaiA. CikA also has a PsR domain, which resembles that of KaiA [45], and oxidized quinone inactivates CikA in a similar manner. Therefore, now CikA and oxidized quinone are added into the standard KaiABC in vitro mixture in order to test whether CikA contributes to a phase delay in response to quinone [46]. During KaiC’s nighttime dephosphorylation phase, KaiA and CikA compete for the same binding site on KaiB. When the bound CikA becomes inactivated by oxidized quinone, it becomes released from the KaiC–KaiB complex, and now the empty spot on KaiB can sequester KaiA, leading to further dephosphorylation. This creates a delay in the oscillation and shifts the oscillation to peak later. In sum, CikA indirectly alters the phosphorylation profile of KaiC by modulating the effective KaiA concentration. Finally, with both CikA and oxidized quinone in the mixture, both a phase advance and a delay of oscillatory KaiC phosphorylation can be generated in response to quinone in vitro, and this gives a more accurate representation of the cyanobacteria’s phase-shifting response to darkness (Figure 4) [47].

It is also worth mentioning that the temperature-compensating property of the cyanobacterial circadian clock may provide a further advantage in survival. This is because the central oscillator should be invariant to minor temperature changes in the environment, whereas almost every chemical reaction speeds up with the increase in temperature and vice versa. It is crucial for cyanobacteria to ensure that the molecular timekeeping is unperturbed. Previously, the standard KaiABC oscillator mixture has been shown to be relatively immune in the biologically meaningful range of temperature between 25 °C and 35 °C, with a Q_10_ value close to 1.0 [13]. In a similar manner, quinone entrainment with the addition of CikA into the mixture also turned out to compensate for temperature [46].

## 3. Physiological Rhythm through the Output Pathway

At dawn, the de novo synthesis of photosystems, ATP synthase, and carbon fixation-related proteins peaks [23,48,49]; at dusk, more than 30% of *S. elongatus*’ transcription activity is shut off [23,49]. It was proposed that cyanobacteria enter this period of dormancy to conserve reducing power and survive under an energy-limited nocturnal setting [50]. Then, how does the central oscillator exert its effect on the metabolic activity of the prokaryote such that not only the right proteins get expressed at the right time but also energy is more efficiently spent?

It has been reported that KaiC activates the autokinase activity of histidine kinase SasA [51]. SasA is known to interact with KaiC directly, forming the KaiC–SasA complex [18]; this activates its autokinase activity. Through a sequence analysis, SasA was found to be composed of a receiver domain and a histidine kinase domain; though, the only structural data available for SasA by this point was that of a thioredoxin-like N-terminal domain [52]. Interestingly, KaiB is known to flip between an inactive (ground) and active (fold-switched) state [18], and the structural similarity between the fold-switched form of KaiB and N-terminal domain of SasA has previously been suggested [18], hinting at their competition for binding KaiC [51,53]. Although KaiC–SasA binding peaks before that of KaiC–KaiB, there is a significant overlap between those binding activities [54], and it is difficult to conclude what accounts for this difference between SasA’s and KaiB’s affinities to KaiC at this point [55].

Then, phosphorylated SasA leads to RpaA phosphorylation, which is a master response regulator that binds more than 134 transcripts [3,56]. The rhythmic change in the RpaA phosphorylation level brings about the activation of gene expression that peaks every 24 h, activating a sigma factor cascade that modulates cyanobacteria’s physiological pattern in a polyphasic manner [57]. This simple output pathway accounts for cyanobacteria’s ability to temporally control the production of proteins that are only needed at a certain time of day, such as enzymes that are needed for photosynthesis or glycogen breakdown [48].

A dogma in circadian clock research was that all timekeeping mechanisms run through the transcription–translation feedback loop (TTFL), where the abundance and gradual degradation of clock proteins renders a periodicity of 24 h. Cyanobacteria emerged as an exception and showed a free-running oscillator component that is embedded in, yet independent from, the TTFL known as the post-translational oscillator (PTO) [12]. This KaiABC-based PTO is suggested to account for the robustness of the cyanobacterial circadian clock, while TTFL is controlled by the oscillatory phosphorylation level of RpaA [58].

Aside from its input functionality, CikA also seems to be involved in other biologically pivotal processes. The cyanobacterial circadian clock controls when it is an optimal time to initiate or halt cell division, and there is a 6 h block of time during the subjective night when cytokinesis is limited. This narrow time frame of preventing reproduction is carefully modulated by the circadian clock such that cyanobacteria stop dividing when the cell is deprived of energy. Δ*cikA* and Δ*kaiB* display elongated cell phenotypes, and this was attributed to the defective clock circuit, which prevents them from initiating cytokinesis [59]. Ectopic expression of CikA made them quickly recover from their elongated state and start dividing. As if it was not prepared for dividing any time soon, Δ*cikA* showed diffuse patterns of FtsZ, which is a bacterial tubulin homolog that becomes localized to the mid-cell as the cell readies for cytokinesis [59]. In relation to this pattern, the cyanobacterial genome undergoes a cyclic chromosomal compaction rhythm in continuous light, and this rhythm is stalled in Δ*kaiC* and Δ*sasA* [60,61], although more work is needed to demonstrate a more direct correlation between the clock components and the rhythmic topological changes of genetic materials [23]. Thus, it was concluded that the circadian clock is involved in transducing the information to the cell regarding the optimal time to spend energy and multiply. By gating cell division and allocating resources for reproduction to be spent in a timely manner, the circadian clock ensures the survivability of cyanobacteria.

Previously, it was argued that CikA is a phosphatase that dephosphorylated phosphorylated RpaA, an activity antagonistic to SasA, which phosphorylates RpaA [55]. This finding clearly demonstrates that CikA is a clock component that harbors both input and output functionality. On the other hand, it was shown more recently that in the in vitro mixture, CikA can phosphorylate RpaA in a temporal manner, even without SasA in the mixture, and this oscillatory phosphorylation of RpaA displayed rhythmic binding to DNA [3,62]. This suggests that CikA may not necessarily antagonize SasA; rather, it might work together as a kinase toward RpaA. Although a detailed analysis is needed in order to pinpoint their true roles in the clockwork, SasA directly binds to KaiC, during the rising phase of KaiC phosphorylation, and CikA’s binding only takes place when the KaiC–KaiB complex forms, during the falling phase [41].

Although a significant portion of the cyanobacterial central oscillator is understood, more recent findings have suggested a necessity for further validation regarding the output components. In addition, it is still a mystery as to why Δ*cikA* displays a shortened period [32], and CikA’s likely bifunctional role makes it difficult to find the real cause of this phenomenon. In conjunction with in vivo, structural, and mathematical data, adding more clock components into the in vitro mixture will provide us more insight into their roles, and it is our goal to elucidate how the intricate clock allowed cyanobacteria to inhabit this small planet for so long.

## 4. Timekeepers in Other Species of Cyanobacteria

Not all cyanobacteria are lucky to have all three oscillator components. Another common species of cyanobacteria, *Prochlorococcus marinus*, managed to keep *kaiC* and *kaiB* but not *kaiA*, choosing KaiBC hourglass-like damped oscillator, which is an alternative to the bona fide circadian clock of *S. elongatus*’ [63]. *P. marinus*’ gene expression rhythm oscillates under a periodic light/dark cycle; however, it dampens when exposed to a constant light environment [63]. *P. marinus’* KaiC stays constitutively phosphorylated even without KaiA [64], and in agreement with this phenomenon, has an A-loop sequence that is different from that of *S. elongatus*. Furthermore, *P. marinus* lacks *cikA* homologs, and this is consistent with the notions that CikA is the newest addition to the circadian clock and that entrainment through quinone-sensing, along with the ability to sustain a free-running rhythm, may have become obsolete, at least in this organism [65]. Different environmental pressures might have slowly forced a modified survival method in this strain, and perhaps, such a high level of clock sophistication was not necessarily paying off.

Another familiar species *Synechocystis* sp. PCC 6803 is known to have a *kaiA* homolog and three *kaiC*s and *kaiB*s [66]. Just like *S. elongatus*, *Synechocystis’* KaiA induces autophosphorylation of KaiC1; however, KaiC2 and KaiC3 stay constitutively phosphorylated without KaiA, as in the case of *P. marinus* KaiC. The rhythmic gene expression in *Synechocystis* also seems to be governed by KaiA–KaiB1–KaiC1, implying its conserved circadian timekeeping function. It would be interesting to find out whether the other oscillator components have any functional variance and significance in the clockworks. Unlike *S. elongatus*, which requires light as a primary energy source, *Synechocystis* is able to grow under continuous dark conditions with only 5 min of light pulse each day in the presence of glucose [67]. It would be intriguing to investigate the biochemical details behind its unique clock circuit that may be entrained by a light pulse application [68].

Originally isolated from a Beppu hot spring in Japan, *Thermosynechococcus elongatus* BP-1 has an optimal growth temperature around 57 °C [69,70]. Its thermophilic nature makes *T. elongatus’* clock proteins suitable for biochemical and structural analyses that can be used for studying those of *S. elongatus* [71,72,73], since *T. elongatus* has all three *kai* genes—*sasA*, *cikA*, and *rpaA*—which are all similar in size and sequence to *S. elongatus*. In addition, *T. elongatus*’ oscillatory gene expression pattern is temperature invariant from 30 to 60 °C [74]. Further work is needed to evaluate how *T. elongatus* not only managed to survive in such a harsh environment but also developed a circadian timekeeping system that is immune to such a wide range of temperatures.

## 5. Concluding Remarks and Future Perspectives

Cyanobacteria rely on the circadian clock to generate a biological rhythm, staying in tune with the 24 h day/night transition in order to optimize survival and reproduction. The temperature-compensated post-translational modification that cycles both in vivo and in vitro maintains a stable oscillation, even without the transcriptional or translational feedback from the cell. At the onset of darkness, oxidized quinone becomes acutely abundant; depending on the timing of the dark pulse application, quinone can inactivate KaiA or CikA. Dissociation of the former results in a phase advance and that of the latter causes a delay in the KaiC phosphorylation rhythm, which is an effect that is equivalent to humans adjusting to a new time zone. The central oscillator transduces the temporal information to SasA and eventually to RpaA; the resultant cyclic RpaA phosphorylation periodically activates gene expression, controlling the physiological and behavioral patterns of cyanobacteria in such a way that they show a competitive advantage as primary producers in various ecological niches wherever sunlight reaches.

Meanwhile, darkness also induces the accumulation of the stress response alarmone, ppGpp, and while this may not have a direct effect on the circadian clock, its different levels of abundance can affect gene expression patterns [75,76]. Moreover, RpaB, another transcription factor, which gets phosphorylated in an antiphasic manner to RpaA phosphorylation, is also dark-induced; a clear link between the circadian clock and RpaB is still missing [5,77]. In sum, more research is needed to move beyond RpaA- and RpaB-dependent transcription such that we can get a better understanding of how the rhythmic KaiC phosphorylation, as well as a light/dark pulse, controls and affects the organism as a whole. This could reveal other biologically crucial mechanisms that are potentially synergistic with the circadian clock’s response to external stimuli.

Cyanobacteria’s billions of years of proliferation and presence on this planet are believed to have transformed Earth’s atmosphere [78]. As time passed by, a common ancestor diverged into different species. However, the conserved *kaiC*s and *kaiB*s within a handful of cyanobacteria, as well as some eubacteria and archaea, suggest that perhaps cyanobacteria’s long survival is attributed to a robust and resettable circadian clock that grants the host a competitive edge in any given environment that oscillates with a 24 h periodicity. As they have much left to show regarding how elaborately this biochemical clock runs, the biological and structural details are just beginning to unfold. We wish to get a glimpse of the functionality of each clock component by reanimating the entire circadian clock in vitro such that we can marvel even more at the hidden complexity of the biological gears of the evolutionary masterpiece.
